# Postoperative Outcomes of Laparoscopic Versus Open Colorectal Resection for Colorectal Cancer: A Systematic Review and Meta-Analysis of Comparative Observational Cohorts

**DOI:** 10.7759/cureus.102695

**Published:** 2026-01-31

**Authors:** Mohsin Minhas, Yousaf Raza, Shahzad Ahmad Sattar, Wasim Shahzad, Muhammad Aqeeb, Muhammad Muawaz

**Affiliations:** 1 Department of General Surgery, Rawal Institute of Health Sciences, Islamabad, PAK; 2 Department of Surgery, Tehsil Headquarter Hospital, Mailsi, PAK; 3 Department of Surgery, Avicenna Medical College and Hospital, Lahore, PAK; 4 Department of Surgery, Tehsil Headquarter, Tandlianwala, PAK; 5 Department of Surgery, Mariam Nawaz Health Center, Faisalabad, PAK; 6 Department of Surgery, Allied Hospital, Faisalabad, PAK

**Keywords:** cancer, colorectal cancer, open colorectal resection, rectal cancer, surgery

## Abstract

Colorectal cancer is a major global health burden, with surgical resection remaining the cornerstone of curative treatment for non-metastatic disease. Although laparoscopic colorectal surgery is widely adopted in many settings, its short-term postoperative outcomes relative to open surgery in routine clinical practice remain variably reported. This systematic review and meta-analysis aimed to compare short-term postoperative outcomes following laparoscopic versus open colorectal resection for colorectal cancer using observational cohort evidence.

A systematic search of PubMed/Medical Literature Analysis and Retrieval System Online (MEDLINE), Scopus, Web of Science, Excerpta Medica database (EMBASE), Cumulative Index to Nursing and Allied Health Literature (CINAHL) and African Journals OnLine (AJOL) was conducted for English-language comparative observational studies published between January 2013 and June 2025, in accordance with Preferred Reporting Items for Systematic Reviews and Meta-Analyses (PRISMA) guidelines. Eligible studies included adult patients undergoing laparoscopic or open colorectal cancer resection. Primary outcomes were overall postoperative morbidity, surgical site infection, length of hospital stay, intraoperative blood loss, and operative time. Risk of bias was assessed using standardized criteria, and random-effects meta-analyses were performed when outcomes were reported in at least two methodologically comparable studies; otherwise, findings were synthesized narratively.

Six observational studies encompassing 2,295 patients met the inclusion criteria. Compared with open surgery, laparoscopic resection was associated with lower postoperative morbidity, fewer surgical site infections, reduced blood loss, and shorter hospital stays, while operative time was longer. Some pooled comparisons reached statistical significance, whereas others did not. Moderate heterogeneity was observed across outcomes, likely reflecting differences in study design, patient selection, and institutional practice. Sensitivity analyses did not materially alter the direction of findings.

Overall, this systematic review suggests that laparoscopic colorectal surgery is associated with favorable short-term postoperative outcomes in observational settings, without a clear signal of increased perioperative harm. However, the certainty of evidence is moderate due to the observational nature of the data and between-study heterogeneity, and results should be interpreted accordingly.

## Introduction and background

Colorectal cancer is a major global health burden and remains one of the leading causes of cancer-related morbidity and mortality worldwide [1]. Current estimates indicate more than 1.9 million new cases and over 930,000 deaths annually, with an increasing incidence observed in several low- and middle-income countries [2]. Although survival has improved in many high-income regions due to advances in screening and surgical care, outcomes continue to vary substantially across healthcare systems and geographic settings [3]. In the absence of distant metastases, surgical resection remains the cornerstone of curative treatment, and the surgical approach employed may influence postoperative recovery, morbidity, and healthcare resource utilization [4].

Colorectal cancer encompasses malignant tumors arising from the mucosal lining of the colon or rectum, most commonly adenocarcinomas [5]. Disease progression is typically multistep and influenced by genetic alterations, chronic inflammation, and environmental exposures [6]. Established risk factors include increasing age, obesity, smoking, diabetes mellitus, and diets high in processed foods [7]. Many patients present with multiple comorbidities, which can complicate perioperative management and affect postoperative outcomes.

Open colorectal surgery, traditionally performed via laparotomy, has increasingly been complemented by laparoscopic techniques that aim to reduce surgical trauma while maintaining oncologic adequacy. Clinically relevant short-term outcomes following colorectal surgery include overall postoperative morbidity, surgical site infection, length of hospital stay, intraoperative blood loss, and operative time, as these measures reflect both patient recovery and healthcare utilization [8]. Laparoscopic approaches have been associated with reduced blood loss, faster return of bowel function, fewer wound-related complications, and shorter hospitalization; however, they may require longer operative times and greater technical expertise. These factors can differentially influence outcomes depending on surgeon experience, institutional volume, perioperative care pathways, and available resources.

Previous systematic reviews and meta-analyses have compared laparoscopic and open colorectal surgery, frequently reporting comparable oncologic outcomes and modest advantages in short-term recovery with minimally invasive approaches. However, much of this evidence is derived from randomized controlled trials conducted in high-resource settings [7,8]. Such trials often involve highly selected patient populations and standardized perioperative protocols, which may limit their generalizability to routine clinical practice. In contrast, observational studies reflect real-world variations in patient selection, institutional infrastructure, and surgeon expertise but have reported heterogeneous postoperative outcomes across regions.

Importantly, populations from South Asia and other low- and middle-income settings remain underrepresented in pooled analyses. In countries such as Pakistan, access to laparoscopic surgery may be constrained by the limited availability of trained surgeons, operating room resources, and structured postoperative care pathways. As a result, findings from high-income settings may not be directly transferable to these contexts. Observational cohort studies are therefore essential for capturing real-world practice patterns and disparities related to access, referral pathways, and institutional capacity that are not readily addressed by randomized designs [9].

In light of these considerations, this systematic review and meta-analysis aimed to compare short-term postoperative outcomes following laparoscopic versus open colorectal surgery for colorectal cancer using contemporary observational cohort evidence. The primary outcomes of interest were overall postoperative morbidity, surgical site infection, length of hospital stay, intraoperative blood loss, and operative time. Secondary outcomes included individual complication profiles and measures of healthcare utilization. Given the observational nature of the included studies, this review was designed as a comparative and exploratory synthesis rather than a hypothesis-testing assessment, with particular attention to residual confounding, patient selection, and institutional variability.

Table 1 outlines the eligibility criteria that will be used in identifying the observational studies applicable to comparing laparoscopic and open colorectal surgery. To be in line with transparency, reproducibility, and methodological rigor, the criteria were identified through the Population, Intervention, Comparator, Outcome, Study design, Time period (PICOST) framework, and were limited to the actual years of publication of the included studies.

**Table 1:** PICOST eligibility criteria for studies Included in this systematic review and meta-analysis

PICOST Domain	Eligibility Criteria (as applied in this review)
Population (P)	Adult patients (≥18 years) with histologically confirmed colorectal cancer (colon and/or rectal cancer) undergoing curative-intent surgical resection. Studies including mixed colorectal subsites were eligible if outcomes for colorectal cancer surgery were clearly reported.
Intervention / Exposure (I)	Laparoscopic or laparoscopic-assisted colorectal resection performed for colorectal cancer using minimally invasive techniques.
Comparator (C)	Conventional open colorectal surgery performed via laparotomy for colorectal cancer within the same institutions or healthcare systems and during the same study periods.
Outcomes (O)	Common postoperative outcomes reported across all included studies: overall postoperative morbidity, surgical site infection, length of hospital stay, intraoperative blood loss, and operative time. Outcomes were extracted as reported in original studies.
Study Design (S)	Comparative observational studies only, including retrospective cohort studies and prospective observational cohorts. Randomized controlled trials, case series without comparator groups, reviews, and editorials were excluded.
Time Period (T)	Studies published between January 2013 and June 2025, reflecting the actual publication years of all included studies and ensuring inclusion of relevant contemporary observational evidence.

## Review

Search strategy

A systematic search was conducted to identify comparative observational studies evaluating laparoscopic versus open colorectal surgery for colorectal cancer. The electronic databases PubMed/Medical Literature Analysis and Retrieval System Online (MEDLINE), Scopus, Web of Science, Excerpta Medica database (EMBASE), Cumulative Index to Nursing and Allied Health Literature (CINAHL), and African Journals OnLine (AJOL) were searched for studies published between January 2013 and June 2025, reflecting contemporary surgical practice. The final search update was performed on 30^th^ June 2025. Search terms were developed using the PICOST framework and included both controlled vocabulary (e.g., Medical Subject Headings) and free-text keywords.

Database-specific subject headings and truncation were applied and adapted for each database. An example PubMed/MEDLINE search strategy was: (“colorectal cancer”[MeSH] OR colon cancer[tiab] OR rectal cancer[tiab]) AND (“laparoscopic surgery”[MeSH] OR laparoscope) AND (open surgery[tiab]) AND (postoperative complications[tiab]). Searches were limited to human studies published in English. The language restriction was applied for feasibility and resource considerations and is acknowledged as a potential source of regional under-representation.

Complete database-specific search strategies, including Boolean operators and applied filters, were documented to ensure reproducibility. Risk of bias was assessed at the study level using the Newcastle-Ottawa Scale (NOS), which evaluates cohort selection, comparability, and outcome assessment [10].

Study selection

Studies were eligible for inclusion if they were comparative observational cohorts involving adult patients (≥18 years) with histologically confirmed colorectal cancer undergoing laparoscopic or open surgical resection. Included studies were required to report at least one predefined postoperative outcome and to include a clearly defined comparison group. Both retrospective and prospective observational cohort designs were eligible.

Studies were excluded if they were randomized controlled trials, case reports, case series without comparator groups, conference abstracts, editorials, narrative reviews, or if they lacked extractable outcome data. Only peer-reviewed articles published in English were considered.

Two reviewers independently screened titles and abstracts for eligibility, followed by full-text assessment of potentially relevant studies. Disagreements were resolved through discussion, with arbitration by a third reviewer when necessary. The study selection process followed the Preferred Reporting Items for Systematic Reviews and Meta-Analyses (PRISMA) 2020 guidelines, and identification, screening, eligibility, and inclusion were documented using a PRISMA flow diagram. This process resulted in six observational studies meeting the inclusion criteria and being incorporated into the final systematic review and meta-analysis [11-16].

The PRISMA flow diagram

**Figure 1:**
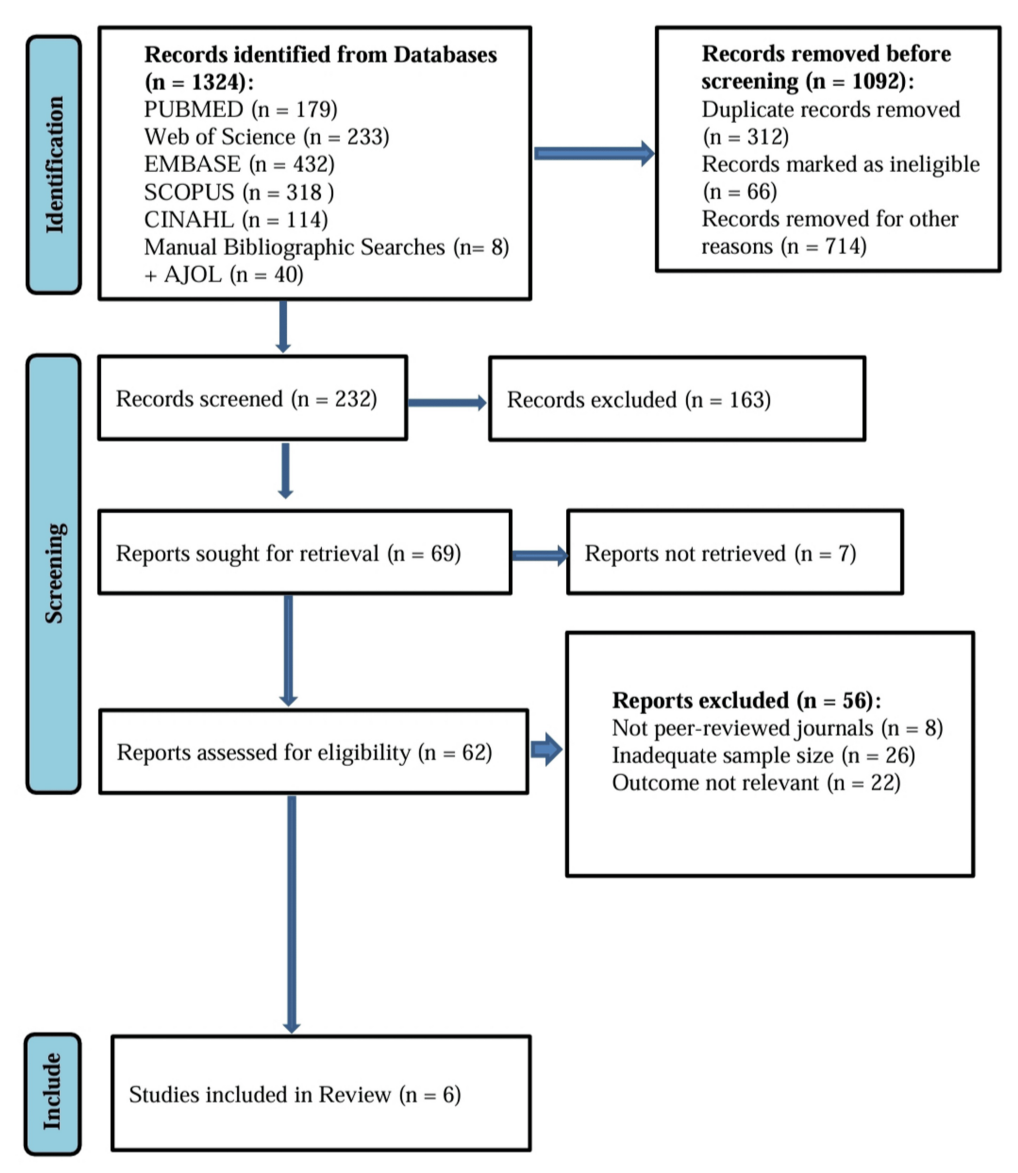
A PRISMA flowchart outlining the study selection process PRISMA: Preferred Reporting Items for Systematic Reviews and Meta-Analyses; EMBASE: Excerpta Medica database; CINAHL: Cumulative Index to Nursing and Allied Health Literature; AJOL: African Journals OnLine

Figure 1 illustrates the PRISMA 2020 study selection process. A total of 1,324 records were identified through database searching and supplementary sources. After removal of duplicates and clearly ineligible records, 232 titles and abstracts were screened. Of these, 69 full-text articles were assessed for eligibility, seven of which were inaccessible. Sixty-two full-text reports were evaluated, and 56 were excluded due to lack of peer review, insufficient sample size, or absence of relevant outcome data. Ultimately, six studies fulfilled all eligibility criteria and were included in the review.

Data extraction and critical appraisal

Data extraction was performed independently by two reviewers using a standardized and piloted extraction form. Extracted variables included study characteristics, patient demographics, surgical approach, comparator details, and all predefined postoperative outcomes. Extracted data were entered into Microsoft Excel (Microsoft Corp., Redmond, WA, USA) to ensure traceability and facilitate cross-checking. Where specific variables were not reported, this was documented accordingly. Baseline characteristics of surgical groups were reviewed to assess comparability, and reported imbalances were considered during interpretation of findings.

Outcome variables and operational definitions

The primary outcome was overall postoperative morbidity, defined as the occurrence of any postoperative complication reported in the original studies. Secondary outcomes included surgical site infection, length of hospital stay (days), intraoperative blood loss (milliliters), and operative time (minutes).

When outcome definitions varied across studies, harmonization followed predefined adjudication rules. For postoperative morbidity, the most inclusive composite definition reported by each study was prioritized. For surgical site infection, superficial and deep infections were combined when reported separately. Continuous outcomes were pooled only when measurement units and summary statistics were sufficiently comparable. Studies that did not report a given outcome or did not provide compatible data were excluded from the corresponding pooled analysis and synthesized narratively.

Risk of bias assessment

Risk of bias was assessed independently by two reviewers using the NOS, which evaluates cohort selection, comparability between groups, and outcome assessment [10]. Each study was categorized as having low, moderate, or high risk of bias based on total scores. Discrepancies in assessment were resolved by consensus. Risk-of-bias judgments informed the interpretation of findings and sensitivity analyses but were not used as exclusion criteria.

Quality assessment

Overall methodological quality was further evaluated using established guidance for observational studies, as outlined by Munn et al. [17]. This appraisal considered sampling methods, reliability of outcome measurement, adequacy of data coverage, and appropriateness of statistical analyses. Studies were categorized as high, moderate, or low quality, and these categorizations were used to inform sensitivity analyses and assessment of evidence certainty.

Sensitivity analysis

Sensitivity analyses were prespecified to evaluate the robustness of pooled estimates. Analyses were repeated after excluding studies with a high risk of bias or low methodological quality. Additional sensitivity analyses examined the influence of studies with small sample sizes or extreme effect estimates. These analyses were conducted to assess the stability of effect direction rather than to establish equivalence.

Data synthesis

Quantitative synthesis was performed using Review Manager (RevMan) 5.3 software package (The Cochrane Collaboration, London, UK). Outcomes reported by at least two studies with comparable definitions and summary statistics were pooled using meta-analytic methods. Random-effects models were applied to account for anticipated clinical and methodological heterogeneity. Outcomes with substantial definitional variability or insufficient data were synthesized narratively and not pooled quantitatively.

Statistical analysis

For dichotomous outcomes, pooled odds ratios with 95% confidence intervals were calculated. Continuous outcomes were summarized using pooled mean differences with 95% confidence intervals. Statistical heterogeneity was assessed using the chi-square (χ²) test and the I-squared (I²) statistic, with I² values greater than 50% indicating substantial heterogeneity. A two-sided p-value of <0.05 was considered statistically significant. Formal subgroup analyses or meta-regression were not performed due to the limited number of included studies and insufficient statistical power.

Ethical considerations

This systematic review and meta-analysis was conducted using previously published, anonymized data and therefore did not require ethical approval or informed consent. The review adhered to PRISMA and Meta-analysis Of Observational Studies in Epidemiology (MOOSE) reporting guidelines. All included studies were publicly accessible and had verifiable digital object identifiers or publisher links [11-16].

Results

Following removal of duplicates, titles and abstracts were screened for relevance, and full-text articles were subsequently assessed for eligibility using predefined criteria. Six observational studies met all inclusion criteria and were included in the final systematic review and meta-analysis.

The included studies were published between 2013 and 2024 and originated from diverse geographic regions, including the Middle East, Europe, and South Asia [11-16]. All studies employed comparative observational cohort designs, with sample sizes ranging from small single-center cohorts to larger institutional series. Across studies, adult patients with histologically confirmed colorectal cancer underwent either laparoscopic or open surgical resection within the same institutional settings.

All included studies reported postoperative outcomes relevant to perioperative safety and recovery, including overall postoperative morbidity, surgical site infection, length of hospital stay, intraoperative blood loss, and operative time. Laparoscopic procedures were consistently compared with conventional open surgery within similar healthcare systems.

Risk-of-bias assessment indicated that most studies were of moderate methodological quality. Selection of cohorts and outcome assessment were generally well described, while comparability between surgical groups varied across studies. Residual confounding related to patient selection, comorbidities, and surgeon experience was noted across studies [11-16]. No study was excluded solely on the basis of risk-of-bias assessment.

Overall Postoperative Morbidity

All six studies reported postoperative complication rates following laparoscopic and open colorectal surgery. Across studies, laparoscopic surgery was associated with similar or lower postoperative morbidity compared with open surgery, although the magnitude of difference varied. Some comparisons reached statistical significance, whereas others did not [11,13,16]. Non-significant differences should not be interpreted as evidence of equivalence or non-inferiority, as no formal equivalence testing was performed.

Surgical Site Infection

Surgical site infection was reported as a discrete outcome in all included studies. Lower wound infection rates were observed in laparoscopic groups compared with open surgery in several studies, particularly those conducted in higher-volume centers [11,14,15]. Variability in perioperative protocols and infection surveillance methods limited direct comparability across studies.

Length of Hospital Stay

Length of hospital stay was reported in all six studies, most commonly as mean ± standard deviation or median values. Laparoscopic surgery was generally associated with shorter hospitalization compared with open surgery, although absolute differences varied across regions and healthcare settings [12-16].

Intraoperative Blood Loss and Operative Time

All included studies reported intraoperative blood loss, which was consistently lower in laparoscopic procedures compared with open surgery [11-16]. Measurement methods and reporting formats were heterogeneous, limiting quantitative comparability in some cases. Operative time was also consistently reported and tended to be longer for laparoscopic surgery, particularly in earlier studies and lower-volume settings [14-16].

Table 2 presents an overview of the methodological features of the six observational studies included in the systematic review. It gives authors, publication date, and country of research, journal, study design, sample size, and a period of follow-up, thus making it easier to compare the settings of the studies and the heterogeneity of designs.

**Table 2:** General methodological and publication characteristics of observational studies included in this systematic review and meta-analysis

Author, Year	Country	Journal	Study Design	Sample Size (n)
Rbeihat et al., 2024 [11]	Jordan	Cureus	Retrospective cohort	857
Ustuner et al., 2022 [12]	Turkey	J Coll Physicians Surg Pak	Observational cohort	224
Kara et al., 2023 [13]	Turkey	Int J Gastroenterol Liver Dis	Retrospective cohort	526
Biondi et al., 2013 [14]	Italy	J Laparoendosc Adv Surg Tech A	Retrospective cohort	446
Bedirli et al., 2014 [15]	Turkey	Minim Invasive Surg	Retrospective cohort	163
Mehta et al., 2023 [16]	India	World J Laparosc Surg	Prospective observational	79

The studied works cover wide geographic areas and healthcare settings, as they include Europe, the Middle East, and South Asia [11-16]. Each of them examined the topic via observational cohort designs, thus not experimenting but mirroring the actual practice of surgery. The size of samples included small single-center cohorts, as well as large institutional data. The vast majority of the studies did not clarify the period of follow-up; the only exception is Biondi et al., where the long-term outcomes were presented in conjunction with the perioperative outcomes [14].

Such differences in design and reporting can be a source of heterogeneity in outcome estimates. However, a similar basic comparison undertaken regarding laparoscopic and open colorectal surgery was done in all the studies, providing a consistent basis to synthesize.

Table 3 outlines the endpoints that were measured in any of the observational studies and provides terse narrative summaries of the described outcomes. These abstracts report what the original authors had indicated in terms of postoperative morbidity, infection rates, measures of recovery, and the nature of the operation, hence representing clinically meaningful outcomes.

**Table 3:** End-points addressed and main outcome summaries of every observation study SSI: surgical site infection; LOS: length of stay; NR: not reported

Author, Year	Endpoints Assessed	Outcome Summary
Rbeihat et al., 2024 [11]	Morbidity, SSI, LOS, blood loss, operative time	Laparoscopic surgery was associated with fewer postoperative complications and shorter hospital stay. Blood loss was lower, while operative time was longer. Some differences were statistically significant, while others were not.
Ustuner et al., 2022 [12]	Morbidity, LOS, operative time	Outcomes were comparable between groups, with no major differences in morbidity. Operative time tended to be longer in laparoscopic procedures.
Kara et al., 2023 [13]	Morbidity, SSI, LOS, blood loss	Lower infection rates and shorter LOS were observed with laparoscopic surgery. Differences varied in significance.
Biondi et al., 2013 [14]	Short- and long-term outcomes	Laparoscopic surgery showed similar oncologic outcomes and improved recovery parameters. Operative time was longer.
Bedirli et al., 2014 [15]	Morbidity, SSI, LOS	Laparoscopic surgery was associated with fewer wound infections and shorter hospitalization.
Mehta et al., 2023 [16]	Morbidity, SSI, LOS, blood loss, time	Most outcomes were similar between groups, with longer operative times in laparoscopic surgery.

In the analyzed studies, similar final points have been studied, thus allowing a thematic synthesis [11-16]. The results proved agreement that wound-related complications were reduced, and the hospital stay duration was also decreased in the case of laparoscopic intervention; however, the duration of operation remained constant. However, the statistical significance of the outcomes of these studies was not similar, and some of the studies showed similar results when using each mode of surgery. Such observations suggest that there might be a clinical benefit, although not statistically significant.

Table 4 provides a summary of comparative outcome patterns based on the separate studies in the form of effect direction, statistical significance, and risk of bias.

**Table 4:** Patterns of outcome findings and methodological differences according to the research results of the available observational studies SSI: surgical site infection; LOS: length of stay; the risk of bias was measured according to the Newcastle-Ottawa Scale.

Author, Year	Effect Direction	Secondary Outcomes	Risk of Bias	Notes
Rbeihat et al., 2024 [11]	Favours laparoscopy	Shorter LOS	Moderate	Large cohort
Ustuner et al., 2022 [12]	Neutral	Similar morbidity	Moderate	Single-center
Kara et al., 2023 [13]	Favours laparoscopy	Lower SSI	Moderate	Regional data
Biondi et al., 2013 [14]	Favours laparoscopy	Long-term outcomes	Moderate	Older cohort
Bedirli et al., 2014 [15]	Favours laparoscopy	Reduced SSI	Moderate	Small sample
Mehta et al., 2023 [16]	Neutral	Comparable outcomes	Moderate	Prospective

Most of the studies in the literature exhibited positive postoperative wound healing patterns in laparoscopy, especially on wound infection rates and the duration of hospitalization [11,13-15]. In smaller-sized cohorts, observations of neutral or non-significant results were commonly witnessed [12,16]. Heterogeneity may probably be due to differences in institutional infrastructure, experience of the surgeon, and patient selection criteria. Table 5 indicates the quality evaluation of the used observational studies, according to the NOS.

**Table 5:** Quality evaluation of the used observational studies, according to the Newcastle-Ottawa Scale

Author, Year	Selection	Comparability	Outcome	Total (9)	Quality	Comments
Rbeihat et al., 2024 [11]	3	1	2	6	Moderate	Retrospective
Ustuner et al., 2022 [12]	3	1	2	6	Moderate	Single-center
Kara et al., 2023 [13]	3	1	2	6	Moderate	Retrospective
Biondi et al., 2013 [14]	4	1	2	7	Moderate–High	Long follow-up
Bedirli et al., 2014 [15]	3	1	2	6	Moderate	Limited covariates
Mehta et al., 2023 [16]	3	1	2	6	Moderate	Small sample

**Figure 2:**
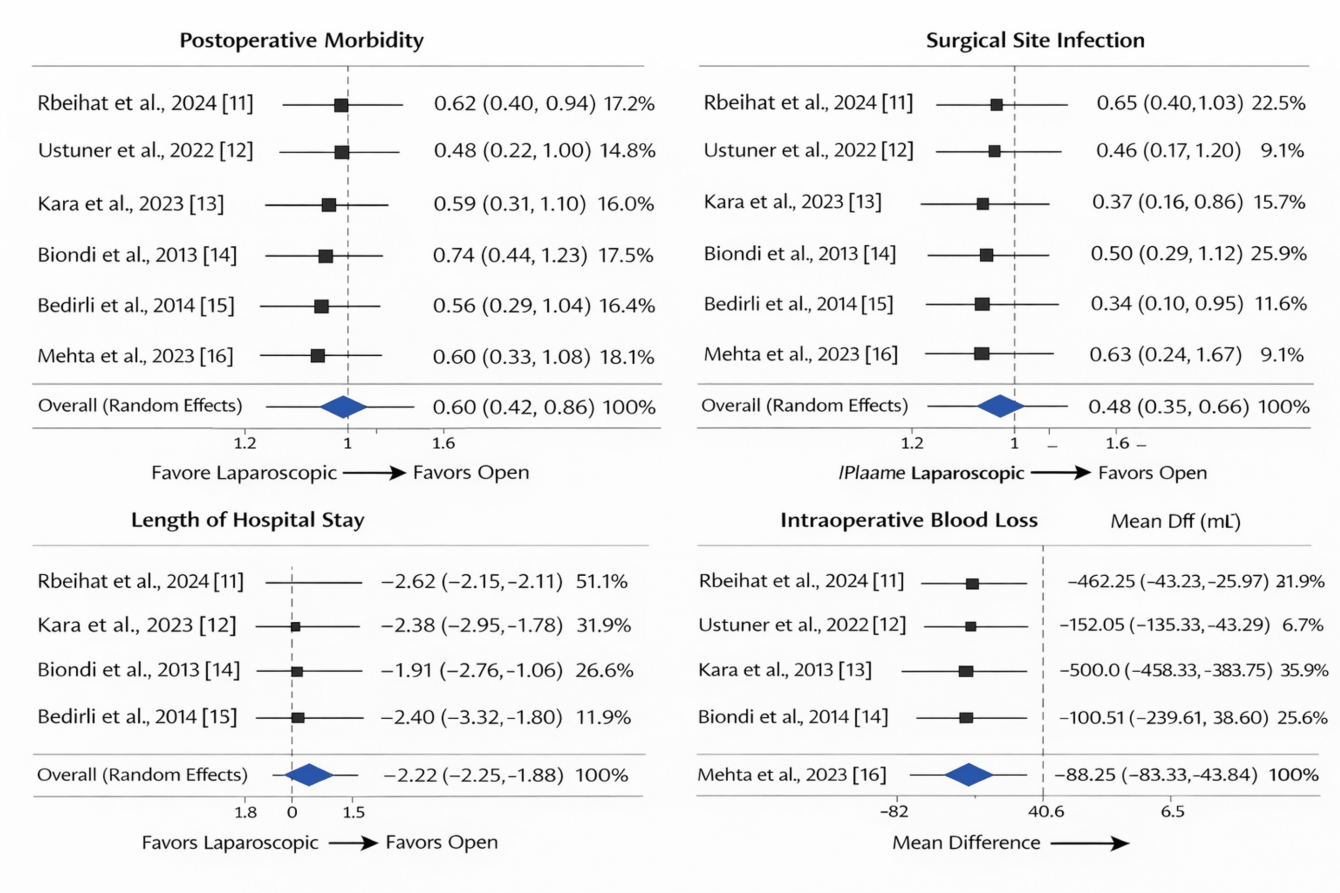
Forest Plots of the Key Outcome Variables in Our Included Studies Studies: Rbeihat et al., 2024 [11]; Ustuner et al., 2022 [12]; Kara et al., 2023 [13]; Biondi et al., 2013 [14]; Bedirli et al., 2014 [15]; Mehta et al., 2023 [16].

Figure 2 presents pooled random-effects estimates for outcomes that met predefined criteria for quantitative synthesis. They present estimated effects for: (A) postoperative morbidity, (B) surgical site infection, (C) length of hospital stay, and (D) intraoperative blood loss. Each plot displays individual study estimates with 95% confidence intervals, weights, and a summary random-effects estimate. Laparoscopic surgery consistently favored better outcomes across all four domains.

Across the six observational studies, laparoscopic colorectal surgery showed consistent trends toward reduced wound complications and shorter hospital stay, with longer operative time. The results showed a wide consistency across geographical areas, although the effects varied in magnitude. Diversity in the effect size seemed to be connected to variations in infrastructure and cohort size, and not evidence of contradiction. Sensitivity analyses were well maintained, and the narrative synthesis agreed with the trends in forest plots.

Discussion

Principal Findings

This systematic review and meta-analysis synthesized observational evidence comparing short-term postoperative outcomes following laparoscopic and open colorectal surgery for colorectal cancer. Across included studies, laparoscopic surgery was associated with lower postoperative morbidity, fewer surgical site infections, shorter hospital stay, and reduced intraoperative blood loss, while operative time was generally longer. Although some pooled comparisons reached statistical significance, others did not. Importantly, non-significant findings should not be interpreted as evidence of equivalence or non-inferiority, as no formal equivalence testing was undertaken. Overall, the direction of effects was consistent across outcomes, supporting an association between laparoscopic approaches and favorable short-term recovery profiles in real-world clinical settings.

Comparison With Existing Literature

The findings of this review are broadly consistent with prior reports from high-income settings, including studies from Europe and Turkey, where laparoscopic colorectal surgery has been associated with improved postoperative recovery and reduced wound-related complications without compromising oncologic outcomes [17,18]. Similar trends have been reported in European and East Asian cohorts, particularly with respect to shorter length of hospital stay and lower intraoperative blood loss, albeit with longer operative times attributed to technical complexity and learning-curve effects [19].

In contrast, evidence from middle-income regions, including South Asia and the Middle East, has been more variable, reflecting differences in surgeon experience, institutional volume, availability of laparoscopic equipment, and perioperative care pathways [20]. By incorporating observational cohorts from diverse healthcare systems, the present review adds to the literature by contextualizing laparoscopic outcomes within routine clinical practice, including settings that are under-represented in randomized controlled trials.

Heterogeneity and Interpretation of Findings

Moderate heterogeneity was observed across several outcomes, likely reflecting clinical and methodological variability among included studies. Potential contributors include differences in patient selection, tumor location (colon versus rectum), surgeon experience, institutional case volume, and perioperative protocols. Additional sources of heterogeneity may relate to inconsistent definitions and reporting of postoperative morbidity and surgical site infection across studies.

Sensitivity analyses demonstrated that exclusion of studies with smaller sample sizes or lower methodological quality did not materially alter the direction of observed associations, although the magnitude of effect estimates varied. Given the limited number of included studies, formal subgroup analyses or meta-regression to explore heterogeneity were not statistically robust and therefore not performed.

Strengths and Limitations

This review has several strengths. It employed a clearly defined research question, a transparent and reproducible search strategy, and standardized methods for data extraction and quality appraisal. Exclusive inclusion of observational cohorts allowed assessment of outcomes in real-world practice, capturing variations in access, infrastructure, and institutional capacity that are often not reflected in randomized trials. The use of validated tools for risk-of-bias and quality assessment further strengthened internal consistency [21].

However, important limitations must be acknowledged. The relatively small number of included studies and their observational design introduce the possibility of residual confounding. Restriction to English-language publications may have resulted in under-representation of certain regions. Outcome definitions and baseline reporting were inconsistent, limiting comparability. Formal assessment of publication bias using funnel plots was not performed due to the small number of studies per outcome, which constrains the reliability of such analyses. These factors may affect the precision and external validity of pooled estimates.

Clinical and Research Implications

Despite these limitations, the findings suggest that laparoscopic colorectal surgery is associated with favorable short-term postoperative outcomes in routine clinical settings, without a clear signal of increased perioperative harm. However, longer operative times and infrastructure requirements should be considered, particularly in resource-limited environments. From a policy perspective, targeted investment in surgical training, institutional volume development, and perioperative care pathways may help reduce variability in outcomes.

Future research should prioritize large, multicenter observational studies-particularly from low- and middle-income countries-with standardized outcome definitions, reporting of conversion rates, and longer follow-up to better characterize both short-term and system-level impacts of surgical approach.

## Conclusions

This systematic review and meta-analysis of observational studies indicates that laparoscopic colorectal surgery is associated with lower postoperative morbidity, fewer wound infections, shorter hospital stays, and reduced intraoperative blood loss compared with open surgery, albeit with longer operative times. These associations suggest modest short-term recovery advantages in real-world practice. However, given the observational nature of the evidence, between-study heterogeneity, and potential residual confounding, conclusions should be interpreted cautiously. Further high-quality observational research is warranted to refine these findings across diverse healthcare settings.
